# Parasite-mediated alteration of behaviour and biomolecular dynamics in a mouse model

**DOI:** 10.3389/fcimb.2025.1574660

**Published:** 2025-09-10

**Authors:** Steven Santino Leonardi, Chin Wen Png, Aye Sandi Bo, Peiyan Wong, Vinaya Rajagopal Iyer, Kevin Shyong-Wei Tan

**Affiliations:** ^1^ Laboratory of Cellular and Molecular Parasitology, Department of Microbiology and Immunology, Yong Loo Lin School of Medicine, National University of Singapore, Singapore, Singapore; ^2^ Healthy Longevity Translational Research Programme, Yong Loo Lin School of Medicine, National University of Singapore, Singapore, Singapore; ^3^ LSI Immunology Programme, Department of Microbiology and Immunology, Yong Loo Lin School of Medicine, National University of Singapore, Singapore, Singapore; ^4^ Neuroscience & Metabolic Phenotyping Core, Department of Pharmacology, Yong Loo Lin School of Medicine, National University of Singapore, Singapore, Singapore

**Keywords:** microbiology, parasitology, gut-brain axis, tryptophan, serotonin, blastocystis

## Abstract

**Introduction:**

Blastocystis is a highly prevalent gut parasite whose pathogenicity remains unclear. Both beneficial and detrimental effects have been observed as a result of Blastocystis infection, including altered gut microbiota, metabolism, and gastrointestinal health. The parasite expresses a modified tryptophanase enzyme known as BhTnaA, which has the unique ability to metabolize indole to tryptophan. Enterochromaffin cells in the gut produce serotonin from tryptophan. These cells are innervated by the vagus nerve, which serves an essential role in mediating bidirectional signaling between the gut and brain. Perturbed serotonin signaling has been associated with disorders linked to gut-brain axis dysfunction, such as IBS and some mood disorders. Our study shows that Blastocystis can use BhTnaA to influence serotonin synthesis by enterochromaffin cells *in vitro* and in a mouse model, and that these effects result in alterations in mouse behaviour.

**Methods:**

We used RIN14B cells as an enterochromaffin cell model to determine whether BhTnaA upregulates serotonin synthesis and associated gene expression. Murine models colonized with multiple Blastocystis ST7 isolates were used to study altered serotonin metabolite levels in the gut. Analysis of mouse behavioral changes was done through the Light Box, Tail Suspension, and Open Field tests.

**Results:**

We demonstrated that the tryptophan produced by BhTnaA upregulates serotonin synthesis in EC cell models. In mice colonized with Blastocystis, increased tryptophan and serotonin levels were observed in the colon, a region of the gut inhabited by the parasites. Behavioral tests showed heightened anxiety in these mice, and a statistical correlation was identified between increases in the metabolites and observed anxiety behaviour.

**Discussion:**

Our study confirmed perturbation of gut tryptophan and serotonin levels by Blastocystis and showed a distinct correlation between this and increased anxiety in colonized mice. This provides a foundation for further investigation into the effects of these parasites on host physiology and the modulation of the gut-brain axis.

**LSID Identifiers:**

*Blastocystis*: urn:lsid:zoobank.org:pub:EAED31FF-9880-4311-9E19-25257588FBB2

## 1 Introduction

*Blastocystis* is an enigmatic genus of highly prevalent protistan parasites. They colonize the colon and caecum of reptiles, birds, and mammals, and are estimated to parasitize over one billion humans worldwide (Boorom et al., 2008; Parija and Jeremiah, 2013). *Blastocystis* are the only parasitic member of the stramenopiles, a group characterized by such organisms as brown algae (*Phaeophyceae*), diatoms (*Bacillariophyta*), and water molds (*Oomycetes*) (Silberman et al., 1996; Derelle et al., 2016). The *Blastocystis* genus is classified into subtypes (STs) rather than species, owing to phylogenetic irregularities that make conclusive molecular assessment of speciation difficult to perform (Gentekaki et al., 2017). There currently exist up to 40 known subtypes, however the validity of some of the more recently-discovered STs is disputed (Stensvold and Clark, 2020; Maloney et al., 2022). *Blastocystis* ST identification is performed through sequencing of the V3/V4 amplicon of 18S ribosomal RNA (Stensvold, 2013). Unless otherwise stated, all results discussed in this article refer to the *Blastocystis* subtype 7, isolate B, referred to as ST7B. Other extant ST7 isolates include C, E, G, and H, all of which were obtained from different patients at Singapore General Hospital in the early 1990s.

The pathogenicity of *Blastocystis* is controversial. Beneficial and detrimental effects have been observed in both *in vitro* and *in vivo* modelling of the parasite’s effects. Multiple laboratories, including our own, have identified improvements in gastrointestinal biodiversity in patients or mice colonized with *Blastocystis* (Audebert et al., 2016; Deng and Tan, 2022). The parasite is considered by some publications to be a component of a healthy gut microbiome (O’Brien Andersen et al., 2016). A positive correlation was observed between *Blastocystis* colonization and lower risks of obesity, cardiovascular diseases, and metabolic disorders (Piperni et al., 2024). On the other hand, *Blastocystis* has been shown to inhibit beneficial gut bacteria *in vitro*, and to induce epithelial damage and mucosal sloughing in pigs and a mouse explant model (Ajjampur and Tan, 2016; Ajjampur et al., 2016). Publications by our lab show that pathogenic *Blastocystis* behaviour tends to originate from ST7 (Yason et al., 2019; Leonardi et al., 2021), and beneficial behaviour from ST4 (Deng et al., 2021; Deng and Tan, 2022; Deng et al., 2023), however insufficient data exists within the literature to confirm whether differences in *Blastocystis* pathogenicity can be fully explained by differences in subtype.

In 2021, we demonstrated that *Blastocystis* synthesizes tryptophan using a modified tryptophanase enzyme (Leonardi et al., 2021). Tryptophanase ordinarily converts the amino acid tryptophan into a signaling molecule, indole. We showed that this enzyme, referred to as *Bh*TnaA (*Blastocystis* tryptophanase), exhibits greater affinity for interactions with indole as a substrate than as a product. We then experimentally confirmed that the *Blastocystis* parasite, when supplemented with indole, does produce tryptophan. The potential implications for this are diverse, as we discussed in a recent opinion article (Leonardi and Tan, 2023). Indole is a major component of intra-microbiome signaling, and is synthesized by a majority of prokaryote species (Tennoune et al., 2022). It has been shown to reduce the virulence of some gut-colonizing pathogens, including *Salmonella enterica* and *Pseudomonas aeruginosa* (Li and Young, 2013). Furthermore, indole has toxic effects on *Blastocystis* ST7 (Ajjampur and Tan, 2016) and, as a result, the parasite could have adapted to metabolizing indole for survival. This change in substrate preference potentially leads to a localized deficiency in indole, which may encourage virulence and growth of pathogenic bacteria in the surrounding environment. Tryptophan is implicated in a vast variety of signaling pathways – it is a precursor to melatonin, niacin, all auxins, kynurenine, and the subject of this article, serotonin (Barik, 2020). Tryptophan is not produced endogenously by mammals, being either synthesized by the gut microbiome or obtained through diet. Any organism capable of altering the tryptophan/indole balance in the gut, even in a localized area, may be able to induce cascade effects elsewhere in the body.

Enterochromaffin (EC) cells are a type of enteroendocrine cell found in the colon and caecum (Wei et al., 2022). Among their many roles, they have the ability to synthesize serotonin from tryptophan in the gut via the enzymes tryptophan hydroxylase (Tph1) and amino acid decarboxylase (Aadc) (Nozawa et al., 2009). These cells are innervated by the vagus nerve, a key component of the gut-brain axis. This nerve performs bidirectional signaling between the gut and brain, transmitting information gathered by EC cells to the brain and returning instructions to the gut that alter conditions within, including inflammation and permeability (Dodds et al., 2022). Perturbed signaling of the vagus nerve has been implicated in the pathophysiology of Irritable Bowel Syndrome and Irritable Bowel Disorder (IBS and IBD, respectively) (Bonaz et al., 2018). Alterations in the tryptophan balance of the gut caused by *Blastocystis* may be able to influence the ability of these cells to produce serotonin.

The neurotransmitter serotonin, or 5-hydroxytryptamine (5-HT), is primarily known for its role in the modulation of mood, cognition, and learning (Buhot et al., 2000; Jenkins et al., 2016; Lu et al., 2019). Approximately 90% of the human body’s serotonin is synthesized in the gut, and regulates gastrointestinal inflammation and the permeability of the gut epithelium (Haq et al., 2021; Banskota and Khan, 2022). Imbalances in serotonin are associated with a wide variety of disorders, including IBS, a highly prevalent idiopathic condition characterized by perturbed gastrointestinal inflammation and permeability (Crowell, 2004; Foley et al., 2011; Jin et al., 2016; Vahora et al., 2020; Bruta et al., 2021). Excess serotonin manifests as a variety of symptoms from diarrhea and shivering to the onset of seizures, while a lack of serotonin causes changes to sleep, mood, and appetite (Correia and Vale, 2022). High levels of serotonin in the nervous system have been linked to depressive symptoms, though the validity of the ‘serotonin theory of depression’ has recently been disputed (Owens and Nemeroff, 1994; Moncrieff et al., 2022).

Colonization by *Blastocystis* is associated with incidence of IBS (Boorom, 2007; Boorom et al., 2008; Jimenez-Gonzalez et al., 2012; Abedi et al., 2022). IBS is also associated with aberrant serotonin levels in the gut (Crowell, 2004; Mawe et al., 2006; Jin et al., 2016; Bruta et al., 2021). *Blastocystis* colonization is further linked to both increased anxiety levels *in vitro*, along with changes to cognition in human hosts (Defaye et al., 2020; Mayneris-Perxachs et al., 2022). Altered bodily serotonin levels are known to influence anxiety symptoms (Alexander et al., 2017; Comai et al., 2020). The perturbation of tryptophan levels and subsequently serotonin synthesis is an enticing potential avenue for the propagation of these processes.

**Figure 1:**
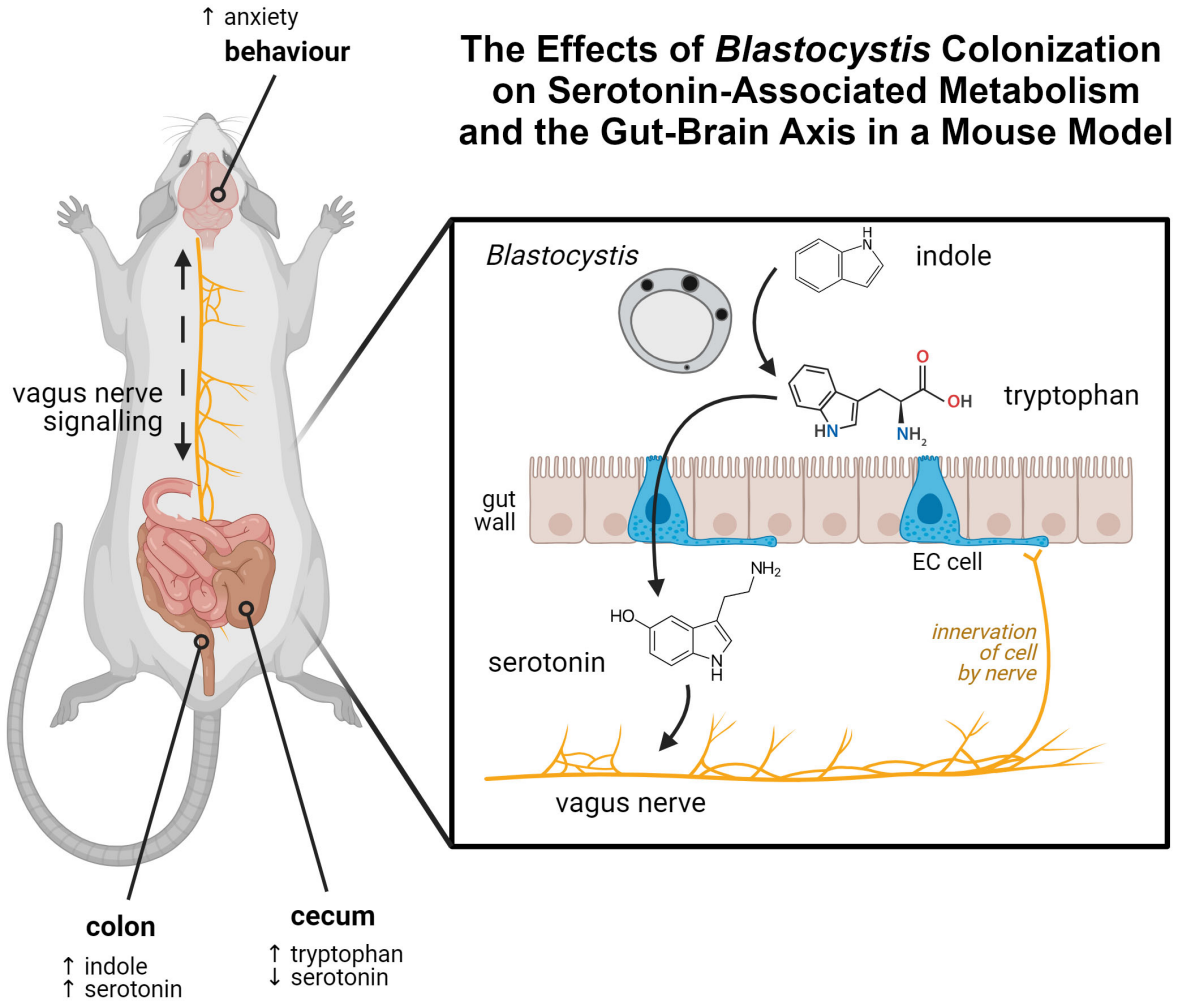
Graphical abstract of the findings of this article. Tryptophan, synthesized by *Blastocystis* from indole in the gut, interacts with enterochromaffin cells in the gut wall. This results in increased synthesis of serotonin by those cells. Serotonin is able to interact with the vagus nerve, which innervates EC cells. A mouse model colonized by *Blastocystis* shows evidence of this process, displaying perturbed indole, tryptophan, and serotonin levels within the gut, along with serotonin-linked behavioural changes. Created in BioRender. Carrera Bravo, C. (2025) https://BioRender.com/f61f123.

We investigated whether *Blastocystis*, using its modified *Bh*TnaA enzyme, is able to sufficiently alter the levels of tryptophan in its environment so as to influence serotonin synthesis by EC cells. This altered serotonin synthesis then perturbs systems throughout the body, including vagal signaling, intestinal conditions, and possibly behaviour. In this paper, we use *in vitro* and *in vivo* modelling systems to investigate the potential consequences of *Blastocystis* tryptophan synthesis within its gastrointestinal habitat. We show that *Blastocystis*-produced tryptophan upregulates serotonin synthesis and associated gene expression in an enterochromaffin cell model. We also show changes to tryptophan and serotonin levels in the gut of a *Blastocystis*-colonized mouse model consistent with our observations in this and our prior study (Leonardi et al., 2021). The outcomes of this project are summarized in **Figure 1**.

## 2 Methods

### 2.1 Blastocystis culture

*Blastocystis* parasites were cultured in 10mL IMDM (Cat. No. 12200036, Thermo-Fisher Scientific, USA) supplemented with CaCO_2_ (3.04g into 1L media) and 10% Horse Serum (Cat. No. 16050114, Gibco, USA). Cultures were contained in individual 14mL round-bottom polystyrene tubes with snap-caps (Cat. No. 352059, Falcon, USA). Anaerobic conditions were achieved by placing the cultures inside an Oxoid AnaeroJar (Cat. No. AG0025A, Oxoid, USA) with an Oxoid AnaeroGen Sachet (Cat. No. AN0025A, Oxoid, USA). All media was pre-reduced in these conditions for a minimum of eight hours prior to inoculation with *Blastocystis*. Cultures were maintained at a consistent 37˚C. Parasites were passaged every 3–5 days, or when the culture media transitioned to a yellow colouration.

### 2.2 Tryptophan assay

5*10^7^ *Blastocystis* parasites were cultured overnight in 1mL IMDM (see above) supplemented with indole, tryptophan, or serine as was necessary. After the culture period, tryptophan levels in media were determined using the Abcam Tryptophan Assay Kit (Cat. No. ab211098, Abcam, USA). Fluorescence intensity was measured at λex = 370nm and λem = 440nm using the Tecan Infinite M200 Pro multimode microplate reader (Tecan Life Sciences, Switzerland, RRID: SCR_019033). Sixteen separate readings were taken from different locations within each well and combined into a single mean measurement. The amount of tryptophan was calculated from a standard curve as per manufacturer protocol. Three independent replicates were performed.

### 2.3 Indole assay

5*10^7^ *Blastocystis* parasites were cultured overnight in 1mL IMDM (see above) supplemented with indole, tryptophan, or serine as was necessary. After the culture period, indole levels were determined using the Assay Genie Indole Assay Kit (Cat. No. BA0046, Assay Genie, Ireland). Absorbance measured at 400nm using the Tecan Infinite M200 Pro multimode microplate reader (Tecan Life Sciences, Switzerland, RRID: SCR_019033). Nine separate readings were taken from different locations within each well and combined into a single mean measurement. The amount of indole was calculated from a standard curve as per manufacturer protocol. Three independent replicates were performed.

### 2.4 Mammalian cell culture

RIN14B (Cat. No. CRL-2059, ATCC, RRID: CVCL_3583) cells were maintained in an environment of 37˚C and 5% CO_2_ in RPMI-1640 (Cat. No. 31800022, Gibco, USA) media supplemented with 10% FBS (Cat. No. A3160401, Gibco, USA). Cells were cultured in T25 and T75 flasks depending on experimental requirements. Cultures were passaged roughly every 3–5 days or upon reaching confluency. Cells were discarded and replaced with cryopreserved samples once passage number exceeded 30.

### 2.5 RT-qPCR

5*10^7^ *Blastocystis* ST7B were cultured overnight in either standard conditions or indole-supplemented conditions as described previously. Parasites were pelleted in a centrifuge run at 6500 x g for six minutes, and the supernatant was then isolated from the samples. RIN14B cells (Cat. No. CRL-2059, ATCC, RRID: CVCL_3583) were seeded on 6-well plates (Cat. No. 657160, Greiner, USA) overnight. The composition of the media of each well varied according to the requirements of the experiment, with a ratio of four parts complete RPMI-1640 (Cat. No. 31800022, Gibco, USA) media to one part requisite supplements, as described below. In the experiments performed in this project, the following supplements were used:

- Control: Fresh, complete *Blastocystis* growth media.
- Indole: Identical to Control, with 1mM indole added.
- Tryptophan: Identical to Control, with 1mM tryptophan added.
- ST7B Supernatant: Supernatant isolated from 24-hour ST7B cultures under standard conditions.
- Indolized ST7B Supernatant: Supernatant isolated from 24-hour ST7B cultures supplemented with 1mM indole.

After 24 hours, the cells were then lysed. The samples proceeded to RNA extraction and cDNA synthesis, as described below, before RT-qPCR was conducted according to the manufacturer protocol of the QIAGEN RT^2^ Serotonin & Dopamine gene expression assay kit (Cat. No. PARN-158Z, QIAGEN, USA). The associated online portal provided by QIAGEN was used to process the data. The qPCR was performed on a CFX96 Touch RT-PCR system (Cat. Nos. 1841100 and 1845097, Bio-Rad, USA, RRID: SCR_018064).

### 2.6 Mammalian cell lysis

RIN14B cells were lysed for RT-qPCR analysis via treatment with RIPA Buffer (Cat. No. 89900, Thermo-Fisher Scientific, USA). Cells were totally aspirated of all media before being washed three times with PBS (Cat. No. UPD8117, Biobasic, Canada). 1 mL RIPA Buffer was added to each well of the 6-well plate containing the cells. This was incubated on ice for 30 minutes, before being centrifuged at 14,000 x g for 15 minutes at 4˚C.

### 2.7 RNA extraction & quantification

RNA was extracted from mammalian cells or using the QIAGEN RNEasy Mini Kit (Cat. No. 74104, QIAGEN, USA). DNAse digestion was performed using the QIAGEN RNAse-Free DNAse Set (Cat. No. 79254, QIAGEN, USA). Nucleic acid purity and concentration was determined via Nanodrop 2000 (Cat. No. ND-2000, Thermo-Fisher Scientific, USA).

### 2.8 cDNA synthesis

Extracted RNA was converted to cDNA via the Bio-Rad iScript cDNA Synthesis Kit (Cat. No. 1708890, Bio-Rad, USA). Nucleic acid purity and concentration was determined via Nanodrop 2000 (Cat. No. ND-2000, Thermo-Fisher Scientific, USA, RRID: SCR_018042).

### 2.9 Serotonin ELISA

These experiments were set up in a similar manner to the RT-qPCR assay protocol above. *Blastocystis* ST7B was cultured overnight under standard or indole-supplemented conditions. This supernatant, along with other supplements as required by the experiment, were added to the media of RIN14B (Cat. No. CRL-2059, ATCC, RRID: CVCL_3583) cells at a ratio of 20% supplements to 80% complete RPMI-1640 (Cat. No. 31800022, Gibco, USA). In addition to the supplement categories described in the RT-qPCR protocol above, the following were also used:

- Inhibited: Grown in 10 µM of the Tph1 inhibitor LP-533401 (Cat. No. HY-15849, MedChemExpress, USA). The concentration used was based on prior research (Watkins-Dulaney et al., 2021).
- Inhibited ST7B Supernatant: Standard condition ST7B supernatant, with LP-533401.
- Inhibited, indolized ST7B Supernatant: Indole-supplemented condition ST7B supernatant, with LP-533401.

Following 24 hours of culture, media was extracted from each well and centrifuged at 8000 x g for five minutes to remove cells and cell debris. Serotonin levels in the media were determined using the Abcam Serotonin ELISA Kit (Cat. No. ab133053, Abcam, USA). Serotonin levels were determined via measuring optical density at 405nm using the Tecan Infinite M200 Pro multimode microplate reader (Tecan Life Sciences, Switzerland, RRID: SCR_019033), followed by the generation of a standard curve from six standards. Provided positive and negative controls were combined with the standard curve to interpolate sample serotonin concentration.

### 2.10 Kynurenine ELISA

Kynurenine levels were determined using the Abbexa Kynurenine ELISA Kit (Cat. No. abx585209, Abbexa, USA). Kynurenine levels were determined via measuring optical density at 450nm using the Tecan Infinite M200 Pro multimode microplate reader (Tecan Life Sciences, Switzerland, RRID: SCR_019033), followed by the generation of a standard curve from four standards. The standard curve was used to interpolate sample kynurenine concentration.

### 2.11 Mouse tissue processing

All mice used in this manuscript were male C57BL/6 (RRID: MGI: 2159769) mice. The gastrointestinal tract was extracted from sacrificed mice and isolated into two separate components: colon and caecum. The tissue samples were placed on ice and transferred to a suitable workstation. Fecal matter was removed from each sample using a forceps, scalpel, and PBS (Cat. No. UPD8117, Biobasic, Canada). Each sample was then transferred to a mortar and pestle (pre-chilled at -80˚C) before being snap-frozen with liquid nitrogen, ground to a powder, and transferred to a 1.5mL Eppendorf tube. The tissue samples were then weighed to determine the quantity of RIPA buffer (Cat. No. 89900, Thermo-Fisher Scientific, USA) required, at 1mL of RIPA buffer (pre-chilled at 4˚C) per 100mg of tissue. If more than 150mg of tissue was present, the tissue powder was transferred to a 15mL Falcon tube. After the addition of the RIPA buffer, the tubes were vortexed for 30 seconds and incubated on ice for ten minutes. A deproteinization step was then performed using the deproteinizing and neutralizing agents contained within the Abcam Tryptophan Assay Kit (Cat. No. ab211098, Abcam, USA). 750 µL of the lysed tissue was combined with 250 µL deproteinization buffer in a fresh 1.5mL tube. This was centrifuged at 13,000xg for five minutes at 4˚C. 570 µL of the supernatant was transferred to a new 1.5mL tube and combined with 30 µL neutralization buffer. The tubes were briefly vented to eliminate the buildup of gas. 200 µL of this solution was then aliquoted into three tubes each for serotonin, kynurenine, and tryptophan plus indole assays, respectively. Indole, tryptophan, serotonin, and kynurenine assays were performed on these samples using an identical methodology to that described prior.

### 2.12 Mouse infection & feces collection

Mice were inoculated with *Blastocystis* parasites over a 21-day period via gavage. Gavage was performed on D0, then three times per week for two weeks for a total of seven gavages, with the final gavage being performed on D14. Mice were gavaged with 100 µL PBS containing 5*10^7^ *Blastocystis*. Control mice were gavaged with 100 µL PBS. From D14 to D21 mice were not gavaged. Each day of the protocol, the cages were placed in a BSC for ten minutes to acclimatize the mice to a BSC environment. This was done to prepare the mice for behavioural experiments in the BSC.

### 2.13 Mouse behavioural tests

All behaviour tests were conducted inside of a BSL2-grade biosafety cabinet. All behavioural data were presented as means ± SD.

### 2.14 Light-dark box test

The light-dark box apparatus composed of two 30x30cm interconnected boxes, one white and one black. A mouse was placed in the light box and allowed to freely explore for ten minutes, while video recorded. At the end of the trial, the mouse was returned to its homecage, and the apparatus was thoroughly cleaned with 70% ethanol (Cat. No. E-0650DF-17, Fisher Chemical, USA). Video recordings were analyzed using CleverSys Inc. TopScan software (RRID: SCR_017141) to obtain the number of times the mouse entered and the total duration spent in the light box.

### 2.15 Open field test

The open field was a transparent acrylic box that was 30cmx30cm in size. A mouse was placed in the box and allowed to freely explore for 30 minutes whilc being video recorded. At the end of the trial, the mouse was returned to its homecage and the arena was thoroughly cleaned with 70% ethanol (Cat. No. E-0650DF-17, Fisher Chemical, USA). Video recordings were analyzed using CleverSys Inc. TopScan software (RRID: SCR_017141) to obtain the distance travelled and average speed of each mouse.

### 2.16 Tail suspension test

A mouse was suspended by having the distal third of its tail taped to the horizontal crossbar of a retort stand that was that suspended at a height of approximately 40cm above the tabletop. The mouse was positioned to ensure that its movements were unrestricted and it faced away from the experimenter. The trial lasted for six minutes and was video recorded. At the end of the trial, the mouse was returned to its homecage and the test area was thoroughly cleaned with 70% ethanol (Cat. No. E-0650DF-17, Fisher Chemical, USA). The recordings were deidentified and scored for the amount of time spent immobile by a blinded external collaborator.

### 2.17 Ethics

IACUC approval was granted under protocol number R24-00060.

## 3 Results

### 3.1 *Blastocystis* tryptophanase activity is in line with prior work

**Figure 2:**
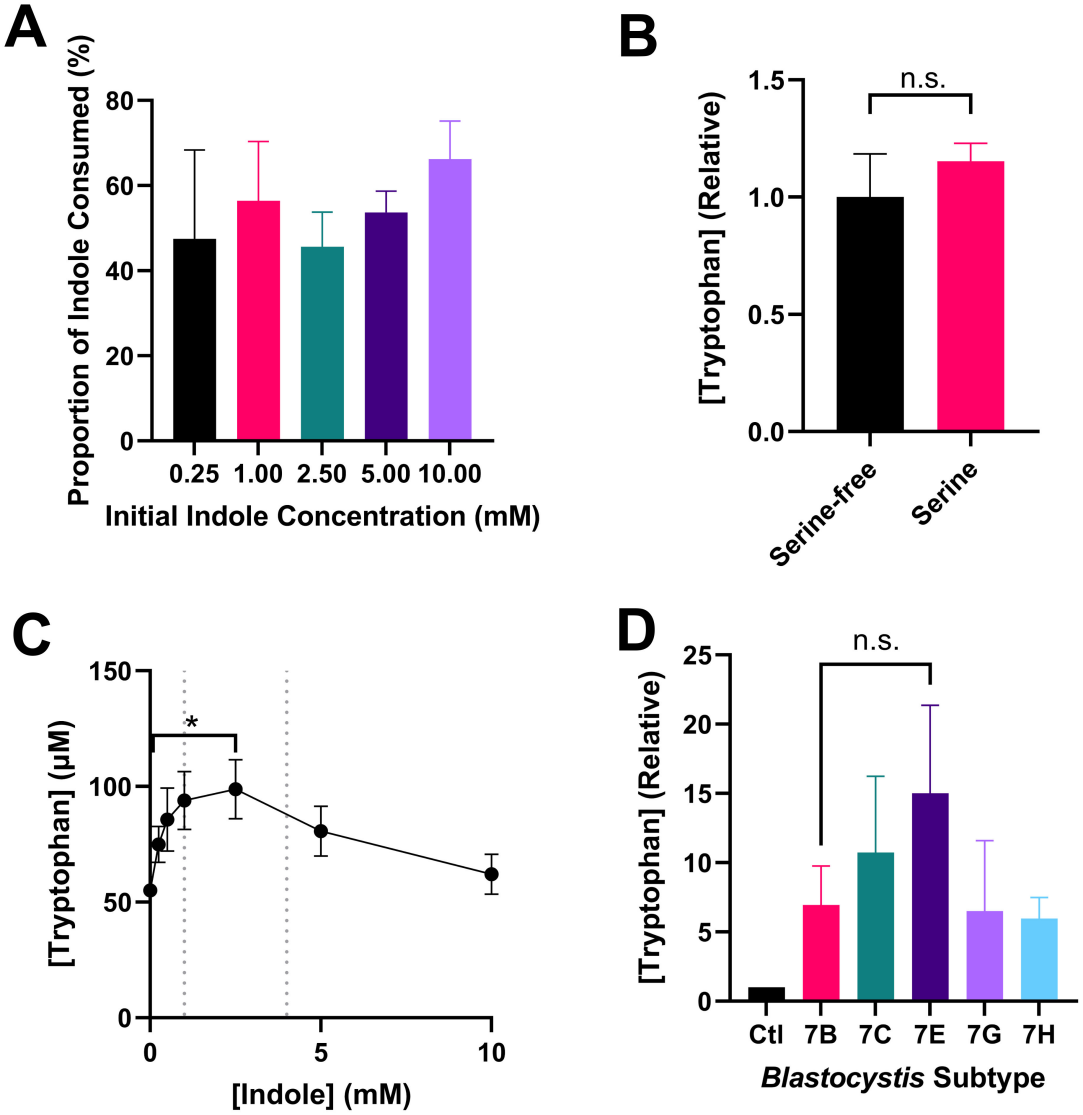
Notable attributes of tryptophan synthesis by *Blastocystis*. **(A)** Proportional consumption of various concentrations of indole by ST7B over 24 hours, n=4 for all samples. **(B)** Effect of serine on tryptophan synthesis by ST7B over 24 hours. Statistical test performed was a Mann-Whitney U test, n=3 for all samples. **(C)** Tryptophan synthesis by ST7B after 24 hour incubation at varying concentrations of indole. Vertical dashed lines represent upper and lower bounds for physiological indole concentration within the human gastrointestinal system. X-axis shows concentration of indole provided to the *Blastocystis*, while the Y-axis shows concentration of synthesized tryptophan. Statistical test performed was a Kruskal-Wallis test, n=3 for all samples. Significance is relative only to the control (leftmost) point. **(D)** Relative differences in the synthesis of tryptophan by various *Blastocystis* ST7 isolates. Control bar represents *Blastocystis*-free PBS supplemented with indole. Statistical significance of the difference between ST7B and ST7E is displayed. Statistical test performed was a series of multiple Wilcoxon tests, n=3 for all samples. n.s. indicates no significance, while * indicates p ≤ 0.05.

**Figure 2A** shows that *Blastocystis* ST7B consumes approximately 50% of available indole, regardless of concentration. This behaviour is observable even at concentrations of 5mM and 10mM, which we previously demonstrated to be toxic to *Blastocystis* (Leonardi et al., 2021).

In our 2023 study (Leonardi and Tan, 2023), we discussed whether the parasites might be able to synthesize tryptophan using the shikimate pathway, along with the *Bh*TnaA enzyme. The shikimate pathway synthesizes numerous molecules including tryptophan. It is present in a wide variety of organisms, including protists like *Blastocystis*. Our prior publication used NCBI BLAST to demonstrate that the *Blastocystis* genome lacked the requisite enzymes to complete the tryptophan synthesis branch of the shikimate pathway. However, we did not assess an alternative tryptophan synthesis, the TrpB enzyme, which uses serine and indole as substrates (Watkins-Dulaney et al., 2021). **Figure 2B** shows that the synthesis of tryptophan by *Blastocystis* is unaffected by the presence of serine, and therefore cannot be based upon the action of TrpB.

To better understand the behaviour of *Bh*TnaA relative to its environment, we assessed the ability of *Blastocystis* to synthesize tryptophan at differing indole concentrations and compared this to the physiological level of indole present within the gastrointestinal system. **Figure 2C** demonstrates that *Blastocystis* ST7B best synthesizes tryptophan in an environment containing between 1 mM and 2.5 mM of indole. The concentration of indole in the human colon is unknown, however the concentration of indole in human feces is between 1 and 4 mM (Darkoh et al., 2015), suggesting that *Bh*TnaA has been adapted for the indole-rich environment of the colon and cecum.

**Figure 2D** is a characterization of the tryptophan-producing behaviour of other *Blastocystis* ST7 isolates in comparison to ST7B. ST7G and ST7H synthesized tryptophan at a rate roughly equivalent to ST7B, while 7C and 7E produced slightly more. The increased tryptophan synthesis observed in those two subtypes was not statistically significant, though ST7E was closest with a p-value of 0.1311.

In our original characterization of *Bh*TnaA (Leonardi et al., 2021), we showed that *Blastocystis* produces a greater amount of tryptophan when supplemented with indole in comparison to a control. This result, while significant, was only weakly so, and was subject to considerable variation. In **Supplementary Figure 1**, we combine that data with further repetitions to emphasize the validity of the original result.

### 3.2 *Blastocystis*-produced tryptophan induces serotonin synthesis in an EC cell model

To determine whether tryptophan synthesized by *Blastocystis* could affect serotonin produced by EC cells, we used the RIN14B *Rattus norvegicus* line as a model. This cell line is the most frequently used EC cell line model in the literature. The cells share a similar gene expression profile with EC cells, and in particular express the Tph1 gene (He et al., 2019; Chapin et al., 2023). Alternate EC cell line models include the KRJ-I and BON cell lines, both of which originate from humans. These cell lines have not been seen in publication since the late 2000’s, and are not maintained by the ATCC (Siddique et al., 2009).

**Figure 3:**
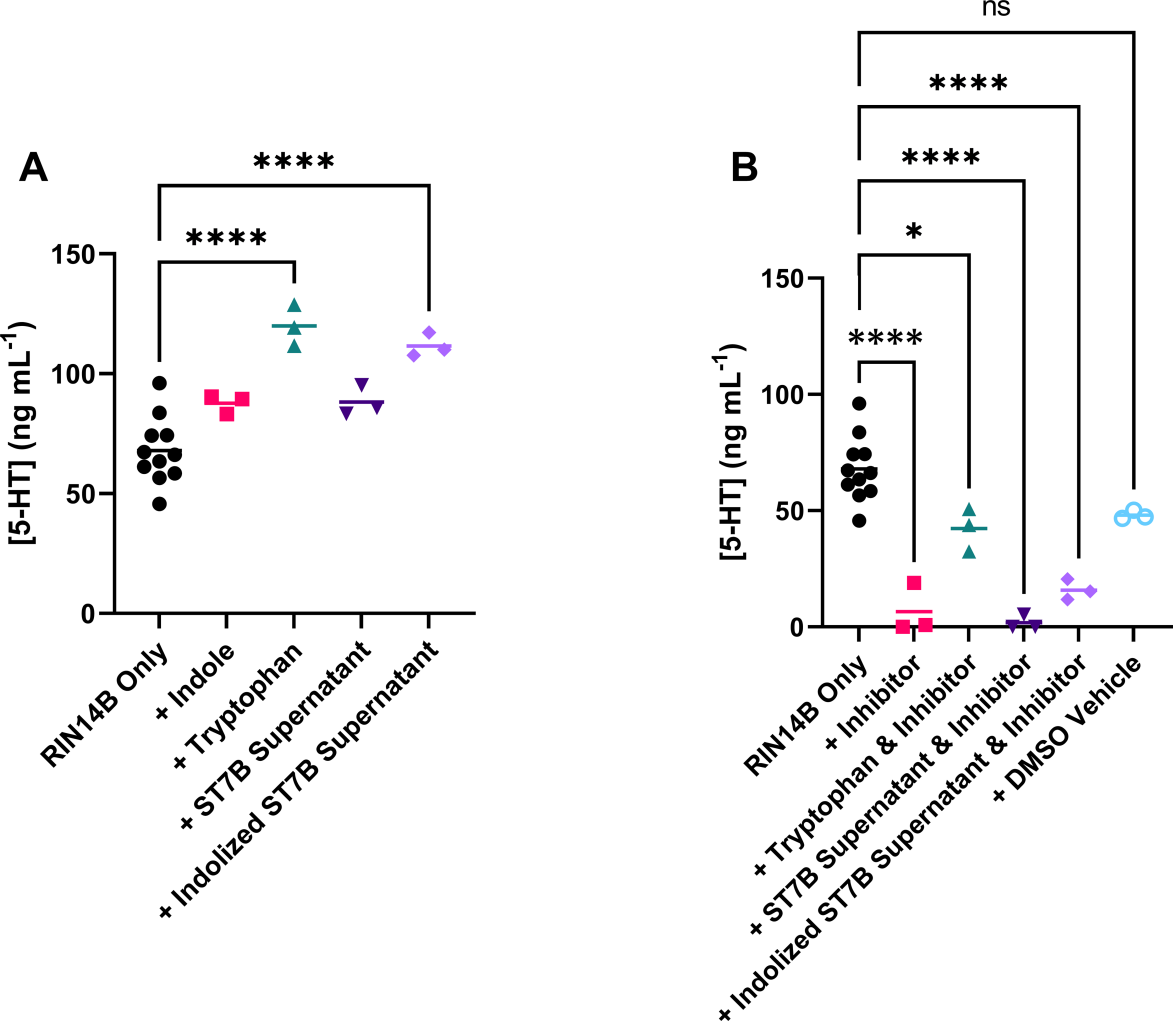
Effects of conditioned *Blastocystis* media on serotonin synthesis by RIN14B cells, measured in concentration of serotonin in media after 24 hours. **(A)** Shows effects without Tph1 inhibitor, while **(B)** shows effects with the inhibitor. Statistical tests performed were ordinary one-way ANOVAs, n=3 for all samples except RIN14B only control, where n=11. Control data in both **(A, B)** is the same. ns indicates no significance, while * = p ≤ 0.05 and **** = p ≤ 0.0001.

**Figure 3** shows the effects of *Blastocystis*-conditioned media on serotonin levels in RIN14B cell media. *Blastocystis* was cultured in tryptophan- or indole-supplemented media overnight. This conditioned media was then harvested and added to a culture of RIN14B cells at a ratio of 1:4 conditioned media to standard RIN14B cell media (for a full explanation, see the Methods section). Our hypothesis was that *Blastocystis*, when supplemented with indole, would convert that indole to tryptophan over the initial incubation period. That tryptophan would then be available to the RIN14B cells to convert to serotonin.

In **Figure 3A**, the addition of indole to the RIN14B culture media does not increase serotonin synthesis. ST7B supernatant also does not produce any change. When *Blastocystis* ST7B is cultured with indole to produce ‘indolized ST7B supernatant’, a statistically significant increase in serotonin synthesis is observed. The positive control – the addition of tryptophan to the culture media – also induces the synthesis of serotonin, as expected. Our hypothesis relies on *Blastocystis*’ effects RIN14B cells being mediated by the rate-limiting step of serotonin synthesis in EC cells: the Tph1 enzyme. To determine whether the effects we observed in **Figure 3A** were dependent on this enzyme, we added the Tph1 inhibitor LP-533401 (Lima et al., 2016) to all experimental groups from **Figure 3A**. **Figure 3B** shows that serotonin synthesis is almost entirely eliminated by the addition of the inhibitor, including when other serotonin synthesis promoters are present such as tryptophan or indolized ST7B. Note that the Control data in both **Figures 3A, B** are identical. The two figures are split for readability.

### 3.3 *Blastocystis*-produced tryptophan upregulates the serotonin synthesis pathway in RIN14B cells

In the previous figure, we demonstrated that *Blastocystis*’ ability to induce serotonin synthesis in RIN14B cells is dependent upon Tph1. With that in mind, we used RT-qPCR to assess whether *Blastocystis*-conditioned indolized IMDM could affect the expression of genes associated with serotonin synthesis, including Tph1. Using the same experimental setup as in **Figure 3**, we extracted RNA from the RIN14B cultures and performed RT-qPCR using the QIAGEN PARN-158Z kit. This kit assesses mRNA expression of 88 serotonin and dopamine-associated genes. The primary purpose of this experiment was to quantify changes in the expression level of Tph1.

**Figure 4:**
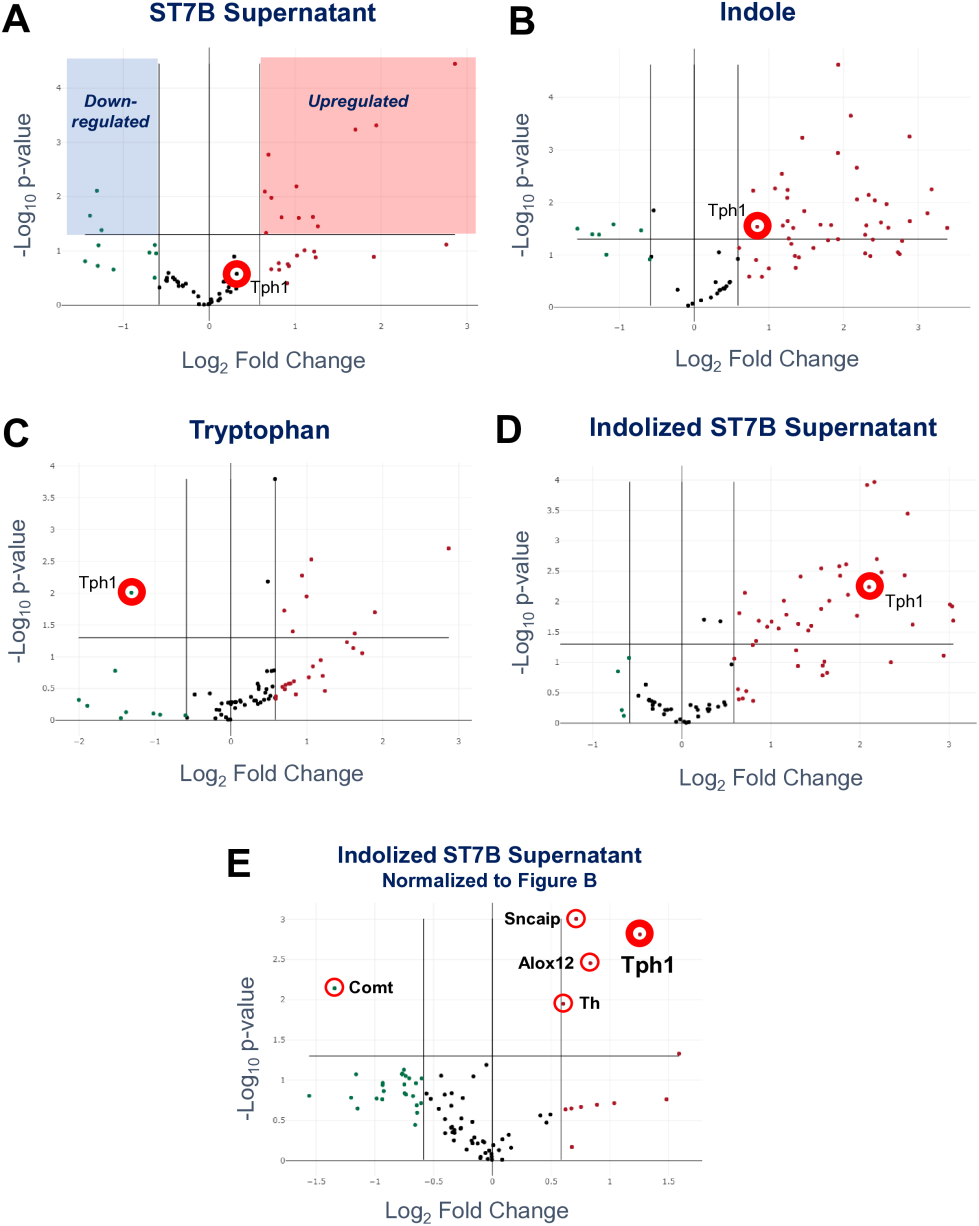
Effects of conditioned Blastocystis media on serotonin-associated gene expression in RIN14B cells. Each subfigure shows the changes induced by Blastocystis media conditioned with a different treatment - ST7B supernatant **(A)**, indole **(B)**, tryptophan **(C)**, and indolized ST7B supernatant **(D)** and **(E)**. Each point represents the expression level of a gene relative to a control dataset, with the exception of subfigure **(E)**, which is normalized to subfigure **(B)**. The x-axis shows fold change in gene expression, while the y-axis shows statistical significance. The horizontal line above the x-axis on each chart shows the p=0.05 cutoff. Points above this line are statistically significant. The three vertical lines represent fold change cutoffs of -1.5, 0, and 1.5, from left to right. Subfigure **(A)** contains highlighted regions demonstrating which regions of each chart contain points that exceed the cutoffs, and are therefore significantly changed by the conditioned Blastocystis media.

**Figure 4A** shows the effects of ST7B supernatant on these 88 genes relative to a control (no treatment) dataset. Tph1 expression was not significantly altered, which aligns with the results shown in **Figure 3**. **Figure 4B** concerns the effects of indole, which was intended to act as a negative control as it did in **Figure 3**. The addition of indole to the RIN14B culture media did produce a small, statistically significant upregulation of the expression of Tph1. This was particularly unexpected, as this treatment did not induce an increase in serotonin synthesis by the cells in the prior experiment. The tryptophan positive control (**Figure 4C**) also produced an unexpected result – Tph1 expression was considerably downregulated, while the corresponding serotonin synthesis in **Figure 3** increased. This is likely a result of the activity of serotonin autoreceptor genes, specifically HTR1A and HTR1B. These genes respond to excess environmental serotonin concentrations by downregulating Tph1 (Best et al., 2010). They are predominantly expressed in neurons, though they are expressed in a small proportion of gastrointestinal cells (Buey et al., 2023). **Figure 3D** shows the effects of indolized ST7B supernatant – here, Tph1 expression is increased, corresponding to the increased serotonin synthesis in **Figure 3**. As the addition of indole was shown to promote Tph1 expression as well, we normalized the results from **Figure 4D** against **Figure 4B**, rather than to the control dataset to produce **Figure 4E**. This figure displays only those genes which were upregulated by the combination of ST7B and indole.

Based on **Figure 4E**, the expression of the following genes was significantly altered by *Blastocystis*-conditioned indolized IMDM: Tph1, Th, and Alox12 were upregulated, while Comt was downregulated. Th and Comt are both components of the dopamine pathway. Th performs dopamine synthesis, while Comt digests dopamine (Eisenhofer et al., 2004). The upregulation of the former combined with the downregulation of the latter suggests an overall increase in dopamine production behaviour. Alox12 is a component of the metabolism of arachidonic acid, which has been implicated in neuronal function, vasoconstriction, and the behaviour of *Toxoplasma gondii* in the host (Witola et al., 2014). Most importantly, Tph1 continued to be upregulated after normalizing for the effects of indole, showing that the combination of *Blastocystis* and indole further promotes serotonin synthesis-associated gene expression.

**Figure 5:**
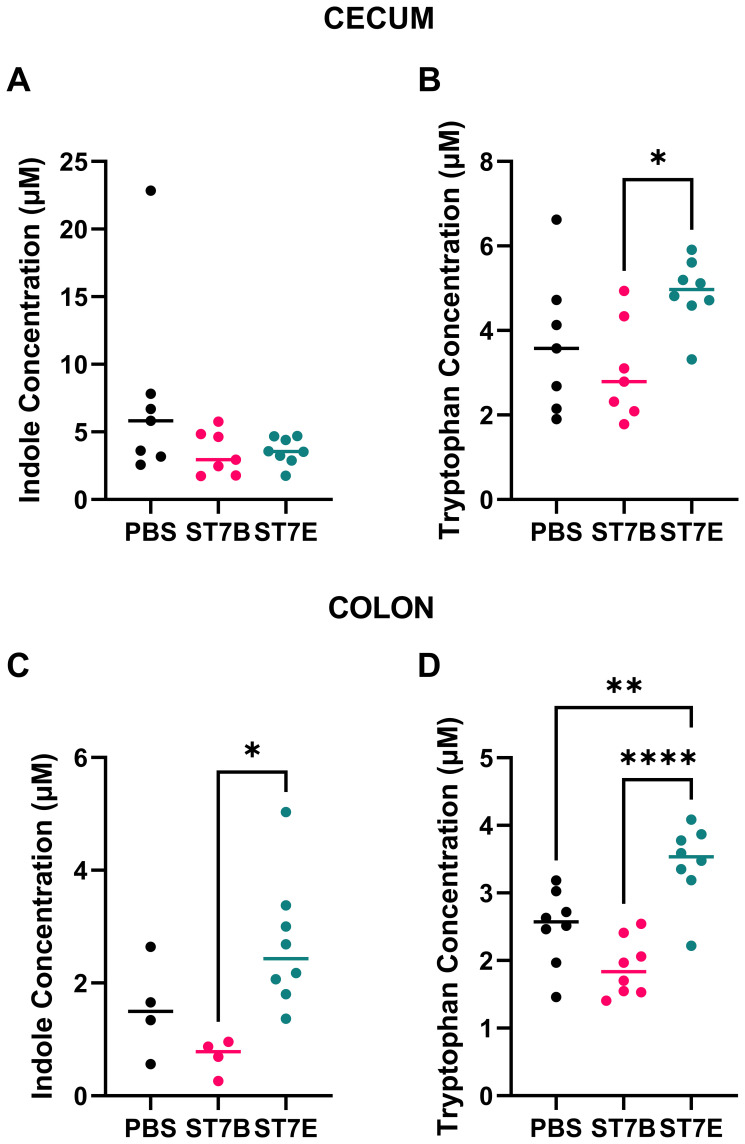
Effects of *Blastocystis* colonization on mouse gastrointestinal tryptophan and indole concentration. **(A, B)** show levels within the cecum, while **(C, D)** show the colon. Coloured horizontal lines show median value. ‘ST7B’ and ‘ST7E’ treatment groups were gavaged with *Blastocystis* 7B and 7E respectively, while the ‘PBS’ control group was gavaged with PBS as a vehicle. Statistical tests performed were ordinary one-way ANOVAs, n=8 for all samples with the exception of **(C)**, where the PBS and ST7B sets are n=4. * indicates p ≤ 0.05, ** = p ≤ 0.01, and **** = p ≤ 0.0001.

We performed Gene Ontology analysis of each of the datasets shown in **Figure 5**. All genes significantly altered by the treatment were analyzed and depicted in **Supplementary Figure 2**. Some notable results: *Blastocystis* ST7B supernatant influences serotonin-linked genes (but not Tph1), even in the absence of indole (**Supplementary Figure 2A**). Neurodegenerative disorder-linked genes, including Alzheimer’s, are upregulated when either *Blastocystis* supernatant or indole is present (**Supplementary Figures 2A, B, D**). The genes upregulated by indolized *Blastocystis* supernatant following statistical compensation for the effects or indole are almost exclusively related to amine biosynthesis, most likely that of serotonin (**Supplementary Figure 2E**).

### 3.4 *Blastocystis* colonization increases tryptophan levels in the mouse colon

Based on the results of our *in vitro* modelling, we assessed whether *Blastocystis* colonization caused similar changes to gastrointestinal metabolites in a C57BL/6 mouse model. We focused on the metabolites indole, tryptophan, and serotonin. We used *Blastocystis* ST7B for all of the *in vitro* experiments thus far, as it is the isolate of *Blastocystis* ST7 that our lab has the most experience with. For the *in vivo* experiments, we also incorporated ST7E as a point of comparison. We selected this isolate as, in **Figure 2D**, we showed that it displayed a level of tryptophan synthesis most divergent from that of ST7B. Three groups of mice containing eight mice each were gavaged three times a week for two weeks: ST7B-colonized, ST7E colonized, and a PBS vehicle. After gavage, the mice were allowed a one-week stabilization period for the equilibration of their gut microenvironment. They were then subjected to a series of behavioural tests (discussed in a later Figure) before being sacrificed. Colonic and cecal tissue was extracted and assessed for metabolite content. **Figure 5** shows that *Blastocystis* colonization did not affect indole concentration in the cecum or colon (**Figures 5A, C**), and did not affect tryptophan concentration in the cecum (5B). Tryptophan concentration appeared raised in the cecum of ST7E-colonized mice, but the increase was not statistically significant. Tryptophan was significantly increased in the colonic tissue of ST7E-colonized mice (**Figure 5D**). Surprisingly, ST7B-colonization did not change levels of tryptophan or indole in any tissue, and in some mice appeared to slightly reduce tryptophan levels.

### 3.5 Gastrointestinal serotonin levels may be increased in ST7E-colonized mice

**Figure 6:**
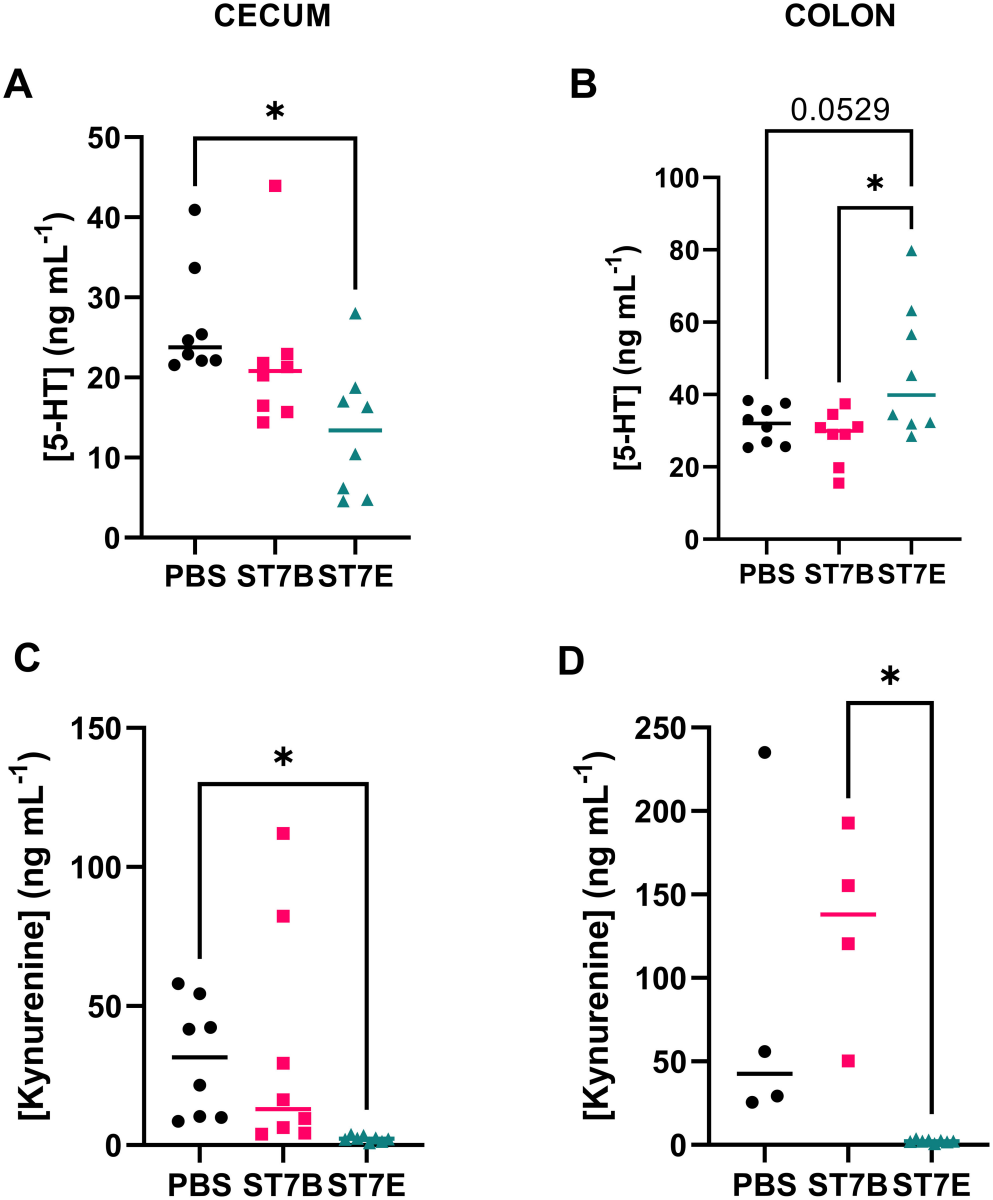
Effects of *Blastocystis* colonization on mouse gastrointestinal serotonin and kynurenine concentration. **(A, B)** show levels within the cecum, while **(C, D)** show the colon. Coloured horizontal lines show median value. ‘ST7B’ and ‘ST7E’ treatment groups were gavaged with *Blastocystis* 7B and 7E respectively, while the ‘PBS’ control group was gavaged with PBS as a vehicle. Statistical tests performed were ordinary one-way ANOVAs, n=8 for all samples with the exception of **(C)**, where the PBS and ST7B sets are n=4. * indicates p ≤ 0.05.

**Figures 6A, B** display the effects of *Blastocystis* colonization on serotonin levels in the cecum and colon of the *in vivo* model. Our hypothesis was that, via the production of tryptophan, *Blastocystis* would induce an increase in serotonin levels in the gut. In the cecum (**Figure 6A**), the opposite is observed – serotonin levels were significantly lower than the control in ST7E-colonized mice. In **Figure 6B**, there is a near-significant increase in colonic serotonin in mice colonized by ST7E, which aligns with the increased tryptophan levels seen in the same tissue in **Figure 5D**. As in **Figure 5**, colonization by *Blastocystis* ST7B did not produce a change in gut serotonin.

Given the link between tryptophan, serotonin, and behaviour, as described in the introduction, we also chose to assess kynurenine levels in the mouse gastrointestinal tissue, which we did not do in our *in vitro* experiments. Kynurenine is another derivative of tryptophan, and is synthesized in multiple sites throughout the body, primarily the liver, brain, and in some parts of the immune system (Badawy, 2017). Tryptophan is trafficked from the gut to these tissues by the bloodstream. Kynurenine can also be produced in the kidneys and gut to a lesser extent – gastrointestinal kynurenine synthesis is driven by EC cells, although these cells do not produce much of the molecule in comparison to other parts of the body. The majority of the effects of kynurenine on the body occur when it is processed into other molecules, such as kynurenic acid, 3-hydroxykynurenine, xanthurenic acid, and quinolinic acid (Okuda et al., 1996; Moroni et al., 2012; Lugo-Huitrón et al., 2013; Jiang et al., 2020). In our assessment of colonic and cecal kynurenine levels (**Figures 6C, D**), ST7E-colonized mice appear to display a nearly complete reduction in kynurenine while ST7B-colonized mice show no effect.

### 3.6 ST7E-colonized mice show heightened anxiety behaviour

**Figure 7:**
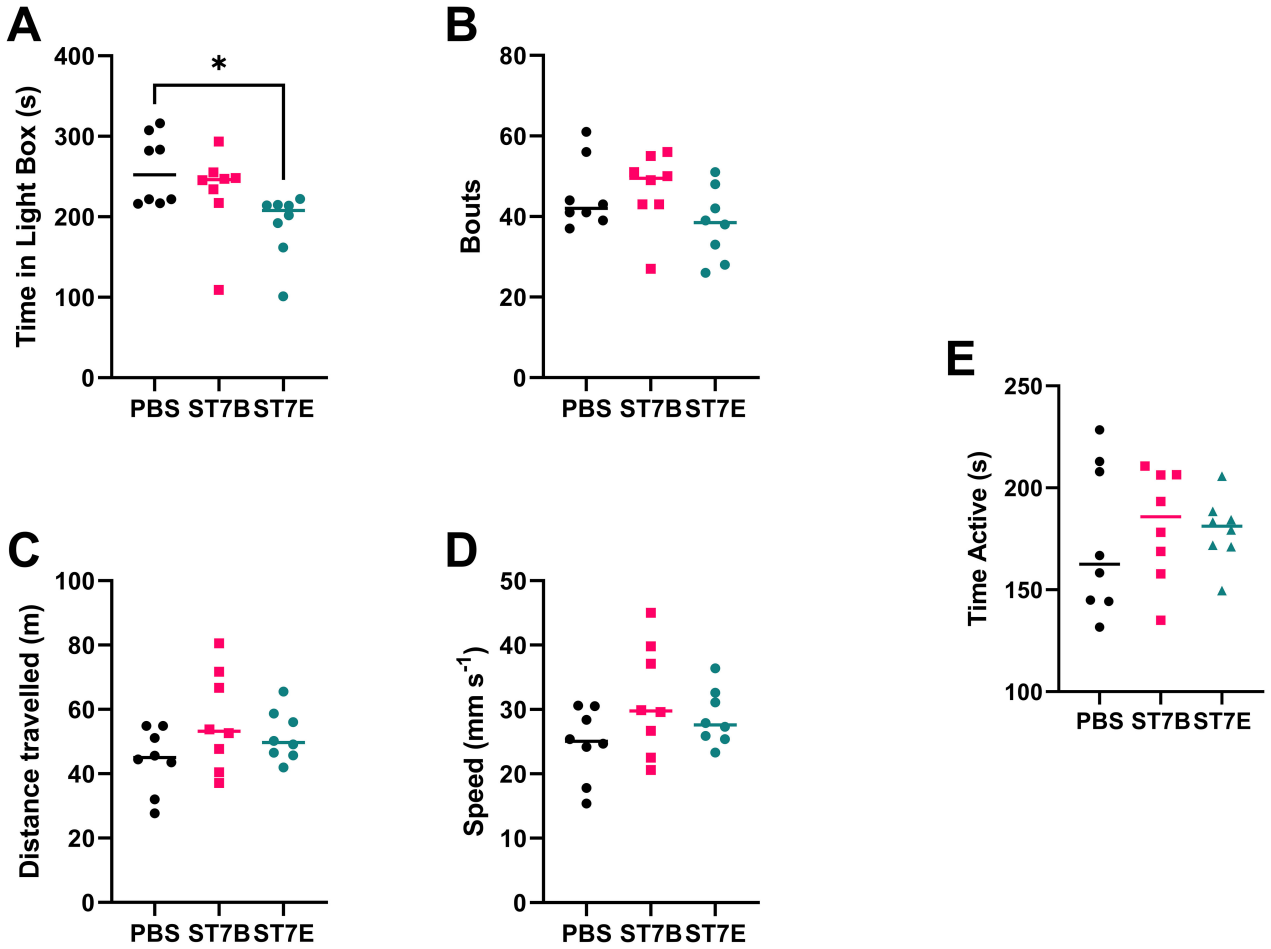
Effects of *Blastocystis* colonization on mouse behaviour. **(A)** shows changes to the duration of time spent in the light box by the mice, while **(B)** shows the number of transits between the light and dark boxes by the mice during the LDB. **(C)** shows the total distance travelled by the mice during the OFT, while **(D)** shows the average speed of the mice during that time. **(E)** shows mouse active time during the TST. Data in subfigures **(A)** through **(D)** was computed via autonomous analysis of recorded videos of the experiments using the CleverSys Inc. TopScan application. Data in subfigure **(E)** was deidentified and analyzed by a blinded external collaborator. ‘ST7B’ and ‘ST7E’ treatment groups were gavaged with *Blastocystis* 7B and 7E respectively, while the ‘PBS’ control group was gavaged with PBS as a vehicle. Statistical tests performed were ordinary one-way ANOVAs, n=8 for all samples. * indicates p ≤ 0.05.

We used the Light-Dark Box (LDB), the Open Field Test (OFT), and the Tail Suspension Test (TST) to characterise changes in mouse behaviour associated with perturbed serotonin levels. The light-dark box test assesses anxiety-like behaviours by measuring the drive to explore a novel environment versus the drive to avoid areas exposed to predation (Arrant et al., 2013), where the less time spent exploring the light box indicates greater anxiety-like behaviours (Malmberg-Aiello et al., 2002; Miller et al., 2011; Acevedo et al., 2014; Wojtas et al., 2022). Here, we showed that the ST7E-colonized mice had a statistically significant decrease in the length of time spent in the light box (**Figure 7A**), with no difference in the number of transitions between the two boxes when compared to PBS mice (**Figure 7B**). This suggests that ST7E-colonized mice showed increased anxiety-like behaviours.

The OFT assesses spontaneous exploratory activity (Stanford, 2007). Here, we used it to demonstrate that changes in exploratory behaviours in the LDB were the result of changes in neurological signaling, including the changes to signaling we expected to see in the case of *Blastocystis*-induced serotonin imbalance, and not due to the differing exploratory behaviours or locomotion abilities of the mice. We did not observe any significant differences in the distance travelled and the average speed across the three groups (**Figure 7C, D** respectively), suggesting that spontaneous exploratory activity was not affected by the colonization of the mice by either isolate of *Blastocystis*.

The TST assesses depressive-like behaviours by placing a mouse placed in a stressful situation for a short duration and monitoring the time spent on struggling to escape and hanging immobile. Mice that show increased depressive-like behaviours would spend more time hanging immobile (Steru et al., 1985; Cryan et al., 2005; Can et al., 2011). **Figure 7E** shows that *Blastocystis*-colonized mice do not exhibit altered depressive-like behaviours.

### 3.7 Gastrointestinal serotonin concentration is linked to anxiety behaviour in ST7E-colonized mice

**Figure 8:**
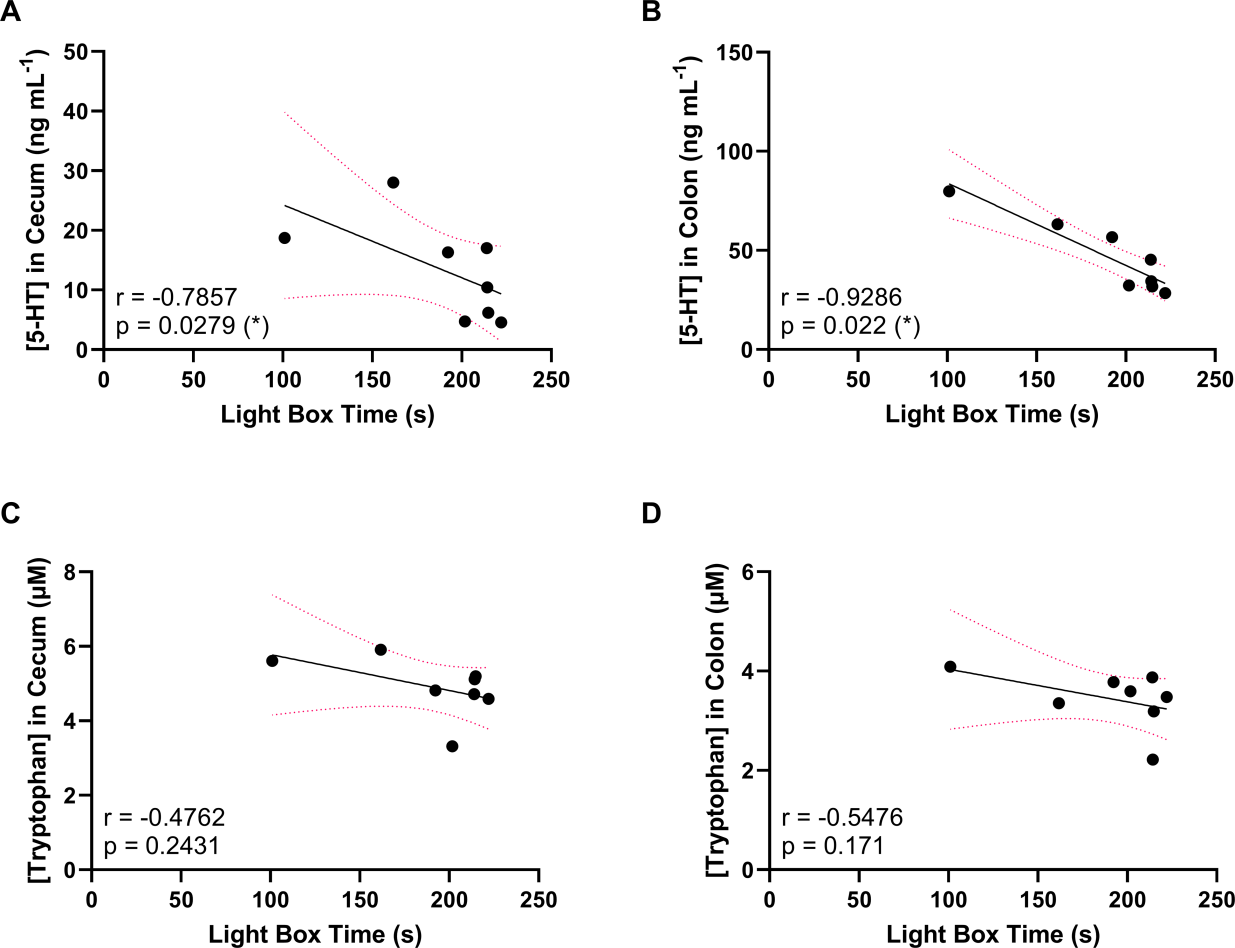
Spearman correlation coefficient analysis of gastrointestinal metabolites against LDB activity in ST7E-colonized mice. Each point represents an individual mouse. **(A, C)** show cecal data, while **(B, D)** show colonic data. Black line shows trend as calculated via linear regression, while dotted pink line shows 95% confidence interval. * indicates p ≤ 0.05.

**Figure 8** shows a series of Spearman correlations performed on data from the ST7E-colonized mice shown in previous figures. The objective of this analysis was to determine whether a link could be established between the anxiety behaviour and the changes to gastrointestinal metabolite concentration observed in these mice. ST7E-colonized mice showed a statistically significant increase in cecal serotonin levels along with an increase in anxiety. **Figure 8A** correlates cecal serotonin concentration with LDB results in these mice. Increased cecal serotonin is negatively correlated with light box time, and is therefore positively correlated with anxiety. Interestingly, when colonic serotonin concentration, which was only elevated at near-statistical significance (**Figure 6B**) is correlated with the LDB data, the relationship is far stronger and is more significant, as shown in **Figure 8B**. **Figures 8C, D** assess whether cecal and colonic tryptophan levels are also correlated with mouse light box time. The direction of the trend is as expected, but the relationships are not statistically significant. Serotonin and tryptophan concentrations are positively correlated in the cecum, but not in the colon (**Supplementary Figure 3**).

## 4 Discussion

### 4.1 *Blastocystis* may deplete indole in localized areas of the gut

The results of **Figure 2A** were unusual, in that the parasites continued to digest indole, even at toxic concentrations. One explanation for this is that it may be more beneficial to the survival of *Blastocystis* to attempt to digest the indole, rather than to simply continue to exist in a high-indole area. As it lacks a flagellum, *Blastocystis* cannot leave such an area in response to chemotaxis. An alternative cause could be a lack of regulatory mechanisms regarding indole uptake, which may be more likely owing to the organisms’ lack of traditional methods of tryptophan synthesis. *Blastocystis* may have simply not yet developed a suitable system for regulating indole uptake. Recently, Wojciech et al. demonstrated that *Blastocystis* produces an indole derivative known as indole-3-acetaldehyde (I3AA) (Wojciech et al., 2023). It may be the case that much of the indole that the parasites deplete is converted to other indolic compounds, which our indole assay protocol did not test for. This may also account for a discrepancy between **Figures 2A, C**, in that the amount tryptophan produced by *Blastocystis* (2C) is considerably smaller than the amount digested (2A). As tryptophan is the starting point for the synthesis of a wide variety of metabolites, the indole digested by the parasites is likely converted into other molecules, including I3AA, during the assay window. Regardless of the explanation, a sufficiently large population of *Blastocystis* in the gut may be able to deplete indole to a sufficient extent that it influences indole’s other functions in that area, such as inter-microbiota signaling.

### 4.2 *Bh*TnaA likely arose as a method for *Blastocystis* to detoxify environmental indole

As shown in **Figure 2B**, serine does not affect *Blastocystis’* ability to produce tryptophan from indole. This confirms that the parasites cannot be using the TrpB enzyme to produce it, as it requires serine to do so. *Bh*TnaA is therefore likely to be the sole mechanism by which *Blastocystis* can produce tryptophan. Our prior work (Leonardi et al., 2021) has shown that *Blastocystis* growth is limited by the concentration of indole that is normally present in its gastrointestinal habitat, thereby providing an incentive for the evolution of an indole-digesting system. Furthermore, **Figure 2C** shows that *Bh*TnaA operates most efficiently within the range of indole concentrations found in *Blastocystis*’ habitat. Taken together, these results suggest that, following the acquisition of a tryptophanase gene in its’ evolutionary past, *Blastocystis* modified this gene specifically to detoxify indole from its environment, via the production of tryptophan.

### 4.3 RIN14B genes upregulated by indolized *Blastocystis* supernatant are involved in a wide variety of pathways

**Figure 3A** showed that indolized *Blastocystis* ST7B supernatant was capable of inducing an EC cell model to synthesize and secrete serotonin. The purpose of **Figure 3B** was to assess whether this change occurred as a result of the rate-limiting step of serotonin synthesis, Tph1. Inhibition of Tph1 prevented serotonin synthesis by RIN14B cells, showing that the effects of *Blastocystis* on EC cell serotonin secretion are dependent on it. Another enzyme in the body also acts as the rate-limiting step of serotonin synthesis, Tph2, though it is only expressed within the brain and is therefore not relevant to this paper (Kanova and Kohout, 2021).

Expression of the Tph1 gene was significantly upregulated by both indole and indolized *Blastocystis* supernatant (**Figures 4B, D**). After normalizing the data in **Figure 4D** against that of 4B to produce **Figure 4E**, Tph1 remained upregulated, suggesting that indolized *Blastocystis* supernatant exerts its own promoting effects on the synthesis of serotonin. Regarding the seeming upregulation of dopamine-related gene expression by indolized ST7B media, as shown in **Figure 4E**, it is important to note that neither EC cells or the RIN14B cell line are known for the synthesis of dopamine. As it was beyond the scope of our project, we did not test for dopamine levels in the RIN14B media as we did with serotonin. It is more than likely that any changes in Th and Comt gene expression in these cells do not translate into the production and secretion of the hormone itself.

The RIN14B cell line is rat-derived. The majority of gastrointestinal serotonin pathways are shared between rodents and humans, particularly the synthesis of serotonin from gastrointestinal tryptophan by Tph1 (Roussin et al., 2024). IBS pathology behaves similarly to humans across both mice and rats (Mawe et al., 2006; Bi et al., 2021). Where rodents differ from humans is in their lack of vagal stimulation-induced emesis (Horn et al., 2013; Yamamoto and Yamatodani, 2018; Zhong et al., 2021) and the ability of their mast cells to synthesise serotonin (Margolis and Pothoulakis, 2009), neither of which are relevant to this study.

### 4.4 RIN14B genes upregulated by indolized Blastocystis supernatant may be associated with neurodegenerative disease

It is worth noting that, in **Figure 4A** (the effects of ST7B supernatant without indole), a number of neurodegenerative disorder-associated genes were significantly upregulated. These include Amyloid precursor protein (App), Synuclein α (Snca), and Synuclein α interacting protein (Sncaip). These genes are involved in the formation of misfolded protein clusters in Alzheimer’s Disease and Parkinson’s Disease (PD) (Nishioka et al., 2020; Tomiyama and Shimada, 2020). Snca and Sncaip, the genes linked to PD, are of particular interest considering recent discoveries regarding the origin of the disease. Braak staging, the most widely-accepted model of the stages of PD (Braak et al., 2003), was only proven in 2019. Braak staging hypothesized that the earliest formation of the neurofibrillary tangles characteristic of PD may occur within the vagus nerve. Kim et al. demonstrated that misfolded α-synuclein fibrils injected into the gut of a mouse are trafficked to the brain, and that the vagus nerve and the expression of the Snca gene are required for this transfer to take place. We consider it highly interesting that *Blastocystis* colonizes regions of the gut inhabited by cells innervated by the vagus nerve, and can upregulate Snca within these cells, as shown in this paper. Gut dysbiosis has been explored as a potential contributor to PD, but no specific member of the microbiome has been identified as a risk factor (Wang et al., 2021). Neurodegenerative disease was beyond the scope of this project, but the potential for an interaction between *Blastocystis* and such disorders is a tantalizing prospect for future research, and may contribute to the growing discourse about the role the health of the gut microbiome can play in the development of neurodegenerative disease.

### 4.5 *Blastocystis* may be capable of influencing Tph1 expression using means other than tryptophan

The increase in Tph1 expression caused by ST7B-conditioned indolized IMDM is independent of the effects of tryptophan. In the tryptophan-supplemented groups of **Figures 3** and **4**, an increase in serotonin synthesis by RIN14B cells was observed, but not an increase in Tph1 expression. Conversely, in the ‘indolized ST7B’ groups, both an increase in serotonin synthesis and Tph1 expression is evident. This suggests that the *Blastocystis*-conditioned media is influencing the expression level of Tph1 through some other means. One possible candidate for this is via *Blastocystis*-secreted extracellular vesicles (EVs). *Blastocystis* ST7B is known to secrete EVs (Leonardi et al., 2022), and the centrifugation we performed to remove the parasites from the conditioned media was not strong enough to precipitate them out of solution. Further research must be done to ascertain the mechanism by which *Blastocystis* can affect the expression of Tph1 and other genes.

### 4.6 *Blastocystis* ST7E recapitulates *in vivo* the effects of ST7B *in vitro*

*Blastocystis* ST7B colonization did not induce any of the changes to mouse gastrointestinal conditions or behaviour we expected based on the *in vitro* data. However, ST7E colonization did cause some of these changes. ST7E colonization resulted in increases in gastrointestinal tryptophan and serotonin (**Figures 5D**, **7B**), along with changes to behaviour that were not present in ST7B-colonized mice (**Figure 7A**). This suggests that distinct isolates of *Blastocystis* can behave very differently within the gut. The increase we observed in tryptophan synthesis by the ST7E isolate may have occurred as a result of this. It is important to note that *Blastocystis* ST7B and ST7E are the same organism according to prevailing 18S rRNA-based *Blastocystis* classification techniques. An explanation for such differences between what is essentially two examples of the same organism is unclear at the current time. While profound genomic differences exist between different subtypes of *Blastocystis* (Gentekaki et al., 2017), intra-subtype differences are poorly characterized. There exists no published genome of ST7E, and only a draft assembly of ST7B (NCBI RefSeq No.: GCF_000151665.1). It is possible that discrepancy between ST7B and ST7E’s *in vitro* and *in vivo* activity may originate from distinctions in the organisms’ interactions with the gut microbiome.

The mice showing a statistically significant decrease in the length of time spent in the light box were the same mice that showed increased gastrointestinal tryptophan and serotonin levels, suggesting a link between these variables further born out in **Figure 8**. Aberrant serotonin levels in particular are associated with changes to anxiety (Comai et al., 2020), though the direction of this relationship is not always clear (Curran and Chalasani, 2012; Marcinkiewcz et al., 2016). A 2016 paper identified a specific pathway within the amygdala of mice that increases anxiety symptoms in response to an increase in serotonin concentration (Marcinkiewcz et al., 2016). The vagus nerve interacts with the amygdala and has been shown to influence neuronal activity within (Alexander et al., 2017). This suggests that, in a mouse model, we should expect to see an increase in anxiety as a response to increased serotonin activity.

The extreme drop in kynurenine shown in **Figures 5C, D** is unexpected, and does not conform to our hypothesis. Further, it is such a large drop that methodological concerns are raised. In controlling for this, we performed the kynurenine assays using multiple different ELISA kits, and all kynurenine assays were completed by the same individual. We find it unlikely that only this specific set of samples experienced some sort of kynurenine-depletion event, and in the absence of an obvious methodological explanation we have chosen to publish the data as-is. Further replication would be required to assess whether ST7E colonization is truly associated with a drop in gastrointestinal kynurenine levels.

Another unexpected result was the decrease in serotonin levels produced by ST7E in the cecum (**Figure 6A**) – this is directly in opposition to our initial hypothesis, and to our *in vitro* results. It may be the case that *Blastocystis* simply behaves differently in different regions of the gut. One contributing factor could be the distinct differences in microbiome between each region, particularly within the gastric mucosa where *Blastocystis* resides (Zhang et al., 2018).

The Spearman correlations in **Figure 8** suggest that our division of the gut analysis into colonic and cecal sections may have been arbitrary. *Blastocystis* ST7E colonization did not result in a statistically significant increase in colonic serotonin, yet it was the level of colonic (and not cecal) serotonin that was associated with the changes in LDB activity displayed by the ST7E-colonized mice. On the other hand, the discrepancy between the cecal serotonin decrease and the colonic serotonin increase does suggest that distinct processes are at play within these regions of the gut. When the average concentrations of tryptophan and serotonin are quantified across both assessed tissues, the statistical significance of the correlations increase, remaining significant at p=0.0107 for serotonin and becoming near-significant at p=0.0694 for tryptophan (**Supplementary Figure 4**). The stronger correlations suggest that a ‘whole-of-gut’ approach may be more preferable or accurate to the separation of the tissues.

### 4.7 *Blastocystis* has the potential to be a potent influencer of the gut-brain axis

Our data does not yet show causal evidence that tryptophan synthesis by *Blastocystis* was responsible for all of the behavioural and gastrointestinal changes observed in our mouse model. To demonstrate this, we would require direct confirmation of increased serotonin/tryptophan levels at the site of *Blastocystis* colonization within the gut of the mice. Nonetheless, this study was able to show that *Blastocystis* subtype 7 can cause gastrointestinal and behavioural changes consistent with serotonin-linked disorders. We establish a consistent throughline from the tryptophan synthesis of the *Bh*TnaA gene characterized in 2021, through its effects on serotonin synthesis by RIN14B cells, to the manifestation of serotonin perturbation and behavioural alteration in *Blastocystis* ST7-colonized mice. These results provide compelling evidence that the effects on anxiety and brain activity seen in prior research (Defaye et al., 2020; Mayneris-Perxachs et al., 2022) may originate from *Blastocystis*-induced perturbation of gut tryptophan and serotonin levels.

## Data Availability

The datasets presented in this study can be found in online repositories. The names of the repository/repositories and accession number(s) can be found below: https://data.mendeley.com/datasets/9jmmpbds5f/3, 10.17632/9jmmpbds5f.3.
