# Supplementary material for: Parasite-mediated alteration of behaviour and biomolecular dynamics in a mouse model
Source: Front Cell Infect Microbiol. 2025 Sep 10;15:1574660. doi: 10.3389/fcimb.2025.1574660 (PMC12457678; doi:10.3389/fcimb.2025.1574660)
Supplement: Supplementary file 1 [file DataSheet1.pdf]

Supplementary Material

1 Supplementary Figures

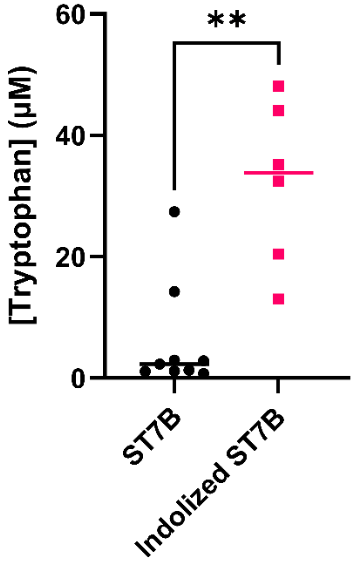

Supplementary Figure 1. Updated ST7B tryptophan synthesis data. Statistical test performed was a Mann-Whitney test, n=9 for ST7B and n=6 for indolized ST7B.

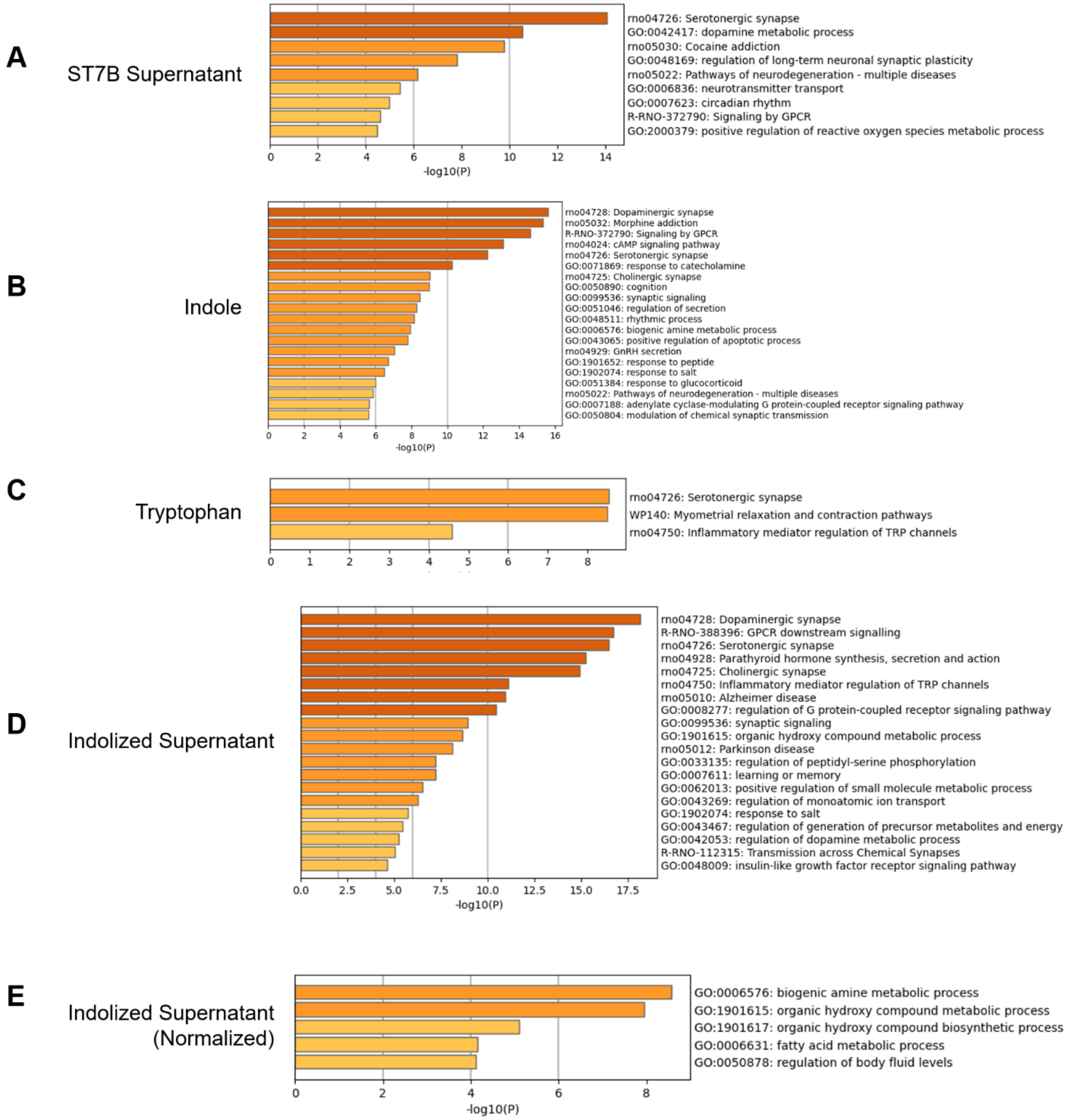

Supplementary Figure 2. Graphical summary of gene ontology analysis of significantly altered genes from the RT-qPCR dataset shown in Figure 5. Subfigures A) through E) correspond to the associated subfigure from Figure 5. The top 20 upregulated terms are shown, where less than 20 terms were statistically upregulated only those are displayed. The size of the horizontal bar shows the statistical significance of the upregulation. Generated using Metascape [86].

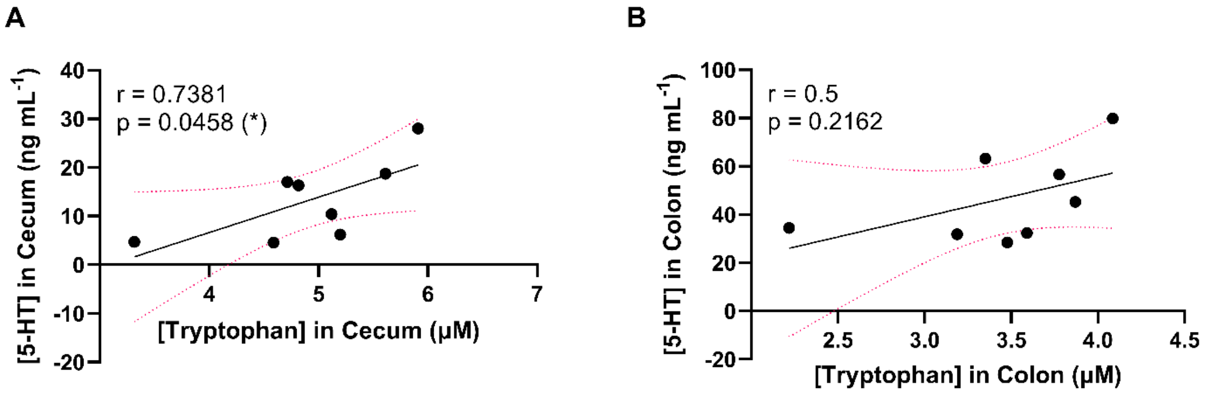

Supplementary Figure 3. Spearman correlation coefficient analysis of serotonin & tryptophan concentration in the colon and cecum of ST7E-colonized mice. Each point represents an individual mouse. Black line shows trend as calculated via linear regression, while dotted pink line shows 95% confidence interval.

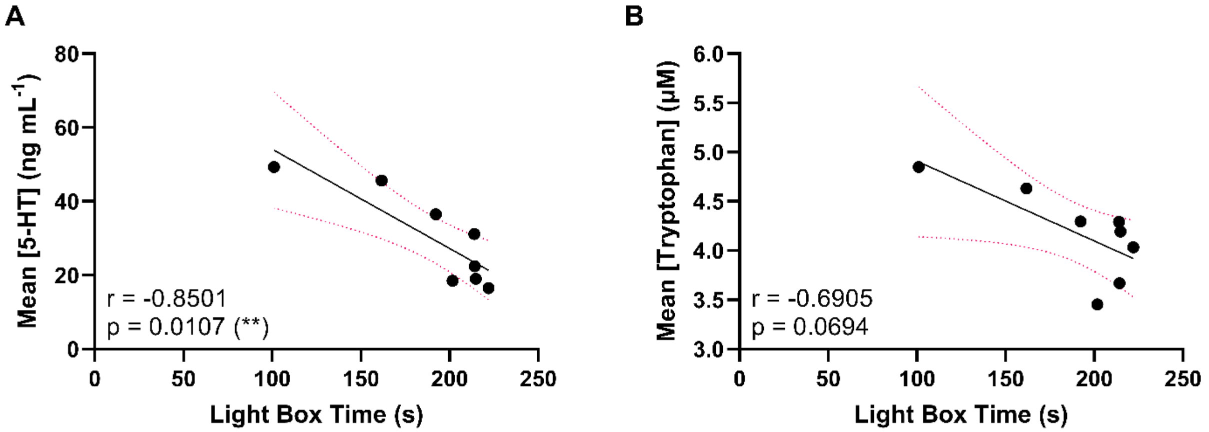

Supplementary Figure 4. Spearman correlation coefficient analysis of mean serotonin & tryptophan concentration in the gut of ST7E-colonized mice. Each point represents an individual mouse. Black line shows trend as calculated via linear regression, while dotted pink line shows 95% confidence interval.
